# gQTL: A Web Application for QTL Analysis Using the Collaborative Cross Mouse Genetic Reference Population

**DOI:** 10.1534/g3.118.200230

**Published:** 2018-06-07

**Authors:** Kranti Konganti, Andre Ehrlich, Ivan Rusyn, David W. Threadgill

**Affiliations:** * Texas A&M Institute for Genome Sciences and Society; † Department of Veterinary Integrative Biosciences; ‡ Department of Veterinary Pathobiology and Department of Molecular and Cellular Medicine, Texas A&M University, College Station, Texas 77843

**Keywords:** collaborative cross, qtl, software

## Abstract

Multi-parental recombinant inbred populations, such as the Collaborative Cross (CC) mouse genetic reference population, are increasingly being used for analysis of quantitative trait loci (QTL). However specialized analytic software for these complex populations is typically built in R that works only on command-line, which limits the utility of these powerful resources for many users. To overcome analytic limitations, we developed gQTL, a web accessible, simple graphical user interface application based on the DOQTL platform in R to perform QTL mapping using data from CC mice.

The utility of model organisms for genetic analysis of biological systems has dramatically increased with the establishment of genetic reference populations. Modern, multi-parental populations specifically designed for quantitative trait locus (QTL) and systems genetics analyses originated with the Collaborative Cross (CC) mouse genetic reference population (Threadgill *et al.* 2002; Threadgill and Churchill 2012). The CC population is derived from eight founder strains, A/J, C57BL/6J, 129S1Sv/ImJ, NOD/ShiLtJ, NZO/H1LtJ, CAST/EiJ, PWK/PhJ, and WSB/EiJ, representing the three major *Mus musculus* subspecies (*M. m. musculus*, *M. m. domesticus*, and *M. m. castaneus*) and which captures 90% of the genetic variation in laboratory mice (Roberts *et al.* 2007). Although the CC has an organized genetic structure (Churchill *et al.* 2004) and is increasingly being used to identify genetic factors controlling a variety of phenotypes from infectious disease and cancer to molecular circuitry (Rasmussen *et al.* 2014; Dorman *et al.* 2016; Venkatratnam *et al.* 2018), genetic analysis of phenotypes using the CC can be challenging due to the multi-allelic structure of the population and complex analytic tools needed to perform analyses (Aylor *et al.* 2011).

Although not a replicable population like the CC, the Diversity Outbred (DO) population was derived from the CC population to increase the recombination load in order to improve mapping resolution for QTL analysis (Svenson *et al.* 2012). To support genetic analysis using the DO population, DOQTL was developed (Gatti *et al.* 2014), which also is increasingly being used for analysis of CC data. DOQTL is an R-based program developed to overcome several analytic challenges of multi-parental populations by implementing an integrated pipeline for haplotype reconstruction, regression modeling to account for kinship, significance thresholds through permutation analysis, and combined association mapping and parental allele-specific tests. Although DOQTL has become the predominant analytic platform for analysis of CC data, it presents a substantial barrier for most biologists with limited computer programming background. Exploiting recent advancements in web framework technologies in R programming, we developed gQTL, which is web application to simplify genetic analyses using data collected from CC mice that will greatly extend the utility of the CC model for a much broader user base.

## Methods

gQTL was implemented using the R Shiny framework (Chang *et al.* 2016), which provides necessary tools for rapid prototyping of interactive web applications. gQTL relies on functions from the DOQTL R package to perform QTL mapping (Gatti *et al.* 2014). Since the CC population has a fixed genetic architecture, associated genotypes and haplotype probabilities for each CC line are stored and loaded into memory in the backend when gQTL is launched. The genotype probabilities for each CC and founder strain were obtained from UNC Systems Genetics data repository (http://csbio.unc.edu/CCstatus/index.py), while the MegaMUGA and GigaMUGA marker set from which the genotypes are determined in the CC was obtained from The Jackson Laboratory data repository (ftp://ftp.jax.org/MUGA/). The user has the ability to choose between either of these marker sets during the submission of the analysis.

### Data availability

The authors affirm that all data necessary for confirming the conclusions of this article are represented fully within the article and its figures. Supplemental material available at Figshare: https://doi.org/10.25387/g3.6453092.

## Results and Discussion

**Figure 1:**
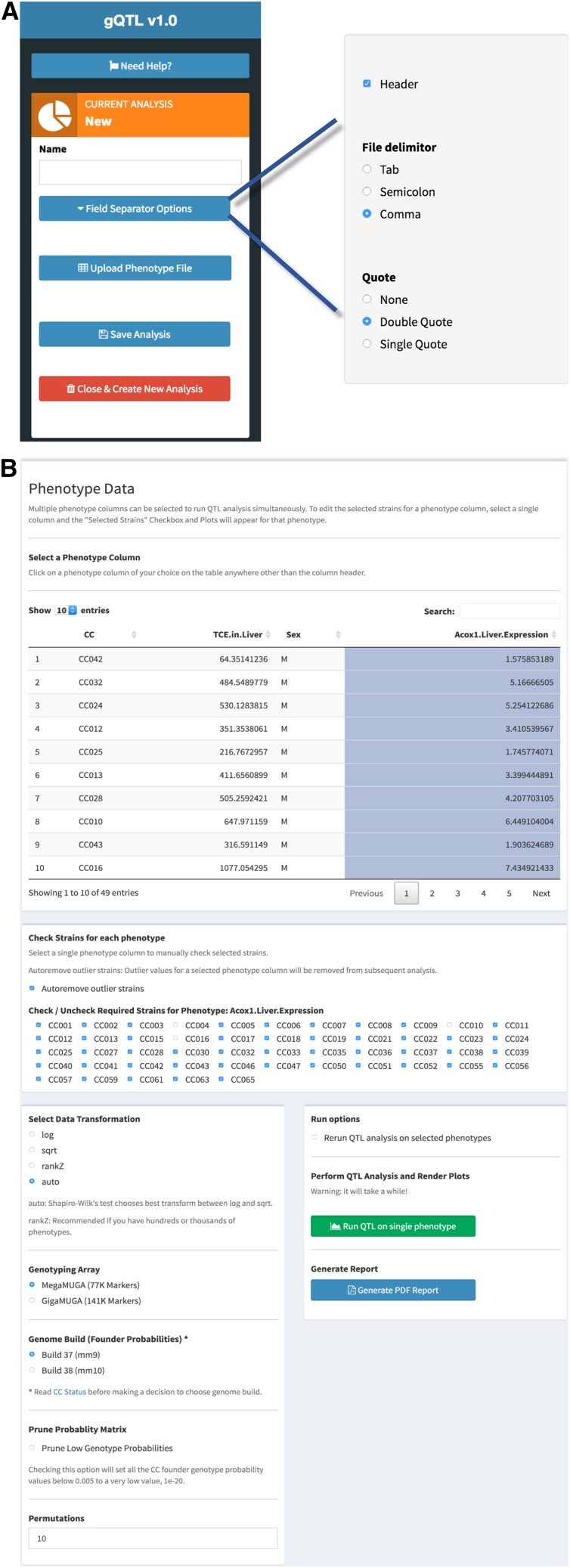
Screen shots of data entry and initial processing. (A) Data loading and file type selection. (B) Uploaded data visualization, outlier selection, and normalization options.

**Figure 2:**
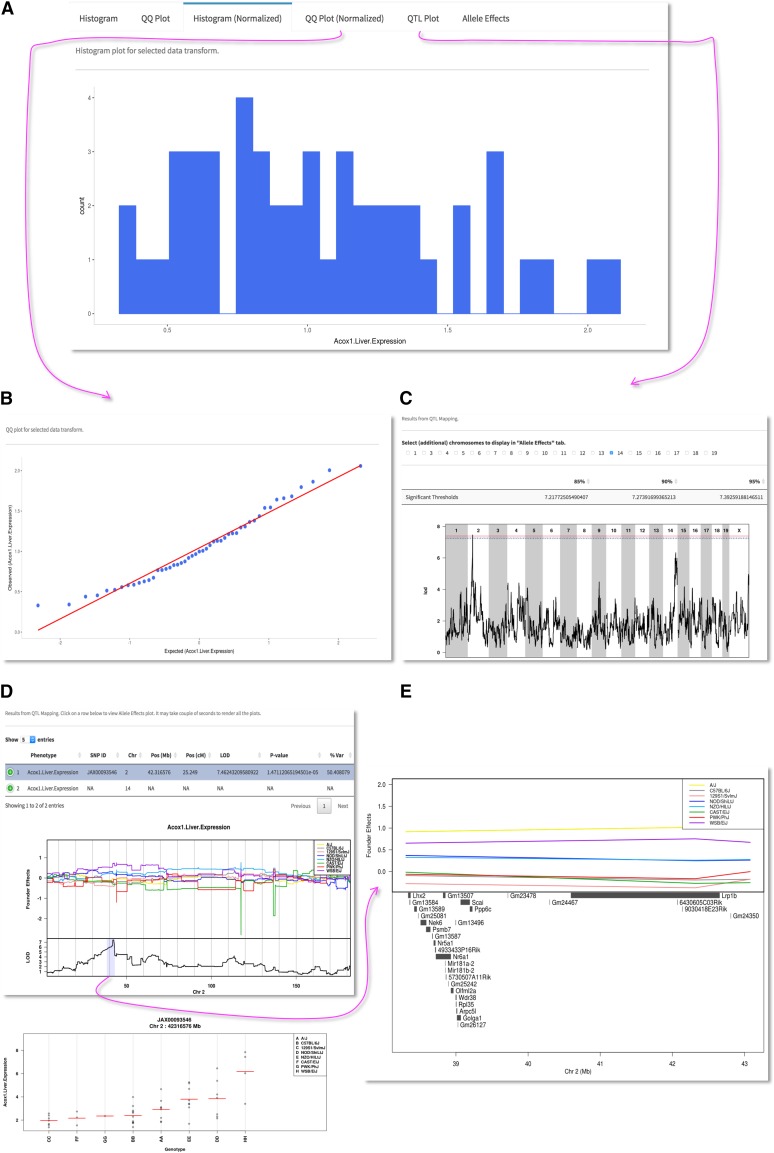
Screen shots of QTL analysis results. (A) Options for data visualization with normalized histogram. (B) QQ plot. (C) QTL plot with threshold levels and locations of significant markers. (D) Allele effect and genotype-phenotype plots. (E) A zoomed version of the significant QTL interval.

After creating a user account, data can be uploaded into a server-side deployment of gQTL, which accepts simple tab delimited or comma separated text files containing a sex identifier and multiple phenotype columns from individual or strain pooled CC data (Figure 1A). At least 3 columns containing Strain (CC), Sex and Phenotype values are mandatory. The CC column can be official or alias names (Supplementary Material, Table S1). Each row can be a line mean or individual mice, sex column should contain M or F, and multiple phenotype columns can be used. In a recent toxicology study, we used the CC population to evaluate the inter-strain variability in oxidative metabolism of trichloroethylene (TCE) and found several QTL controlling tissue TCE levels and expression of specific genes using DOQTL (Venkatratnam *et al.* 2017); datasets from this project are used here to illustrate simplicity of gQTL (Supplemental Material, Table S2). After uploading the data file, users can remove outliers, normalize the data and perform QTL mapping. Uploaded data are presented as a table, wherein specified phenotype columns can be selected for analysis (Figure 1B). Data from specific CC strains for each phenotype can be manually removed using simple check boxes, or automatic outlier removal can be selected. Trait outliers are detected using the standard boxplot outlier rule, 1.5 × interquartile range (IQR) (Tukey 1977). Multiple data transformation choices (log, sqrt, rankZ) are available for user selection, or an automated transformation selection feature can be specified that uses the Shapiro-Wilk test of normality to determine the optimal transformation between log and sqrt (Shapiro and Wilk 1965). For a selected phenotype column, data quality plots, including raw and normalized histogram and QQ plots, are displayed (Figure 2). Finally, individual or multiple phenotype data columns can be submitted to the server for QTL mapping. Significance thresholds are determined through permutation analysis using a user-specified number of permutations (Churchill and Doerge 1994). QTL mapping with 1000 permutations typically takes about 5 hr to finish due to the fact that DOQTL runs on a single core; future implementations will transition to multiple cores. E-mail notifications keep the user informed on the current state of the job(s) running on the server. Each user account can store up to seven different analyses for later revisiting and re-submission of QTL mapping jobs with different parameters.

After the analyses are complete, QTL results can be explored using the web application (Figure 2; Gatti *et al.* 2014). Linkage plots are displayed along with permutation determined LOD scores for the 85, 90 and 95% significance threshold levels. Chromosome-wide, CC founder strain-specific allele effect plots are automatically generated for any locus reaching significance that shows the marker ID with the maximal LOD and its location in cM and Mb coordinates on Build 37 (mm9) or Build 38 (mm10) depending on marker set selected, as well as Mb coordinates of the confidence interval based on a 95% Bayesian credible interval (Sen and Churchill 2001). Higher resolution images of the 95% intervals can be selected that show underlying gene annotations. Other chromosomes that may contain regions of interest but not reach at least 85% significance can be manually selected to generate additional chromosome-specific allele effect plots. For those loci reaching at least 85% significance thresholds, phenotypes for each CC sample is also plotted by genotype to visualize those genotypes driving the QTL signal. A comprehensive PDF report is automatically generated for archiving (Supplemental Material, Figure S1). Additionally, a ZIP archive containing the PDF report along with publication quality PNG figures at 600 dpi can be downloaded.

gQTL v1.0 provides an easy to use graphical user interface for QTL mapping analyses of studies in CC mice with the upload of quantitative phenotype data collected in CC mice being the only input required from users. We plan to extend the application to include the ability to use phenotypes from CC Recombinant Inbred Intercrosses (CC-RIX) in subsequent version releases (Zou *et al.* 2005).

## Web Resources

The web application is freely available at: https://genomics.tamu.edu/gqtl. A built-in help menu exists on gQTL with instructions on setting up user accounts, uploading phenotype data files, inspecting phenotype data, running QTL analysis, viewing QTL analysis results and generating reports of QTL results. The source code, from the original developers (Gatti *et al.* 2014), for the underlying DOQTL package is available at GitHub (https://github.com/dmgatti/DOQTL).
